# Quantifying Interpreting Types: Language Sequence Mirrors Cognitive Load Minimization in Interpreting Tasks

**DOI:** 10.3389/fpsyg.2019.00285

**Published:** 2019-02-18

**Authors:** Junying Liang, Qianxi Lv, Yiguang Liu

**Affiliations:** Department of Linguistics, Zhejiang University, Hangzhou, China

**Keywords:** interpreting types, language sequence, frequency motif, position-dependent properties, cognitive load minimization

## Abstract

Most interpreting theories claim that different interpreting types should involve varied processing mechanisms and procedures. However, few studies have examined their underlying differences. Even though some previous results based on quantitative approaches show that different interpreting types yield outputs of varying lexical and syntactic features, the grammatical parsing approach is limited. Language sequences that form without relying on parsing or processing with a specific linguistic approach or grammar excel other quantitative approaches at revealing the sequential behavior of language production. As a non-grammatically-bound unit of language sequences, frequency motif can visualize the local distribution of content and function words, and can also statistically classify languages and identify text types. Thus, the current research investigates the distribution, length and position-dependent properties of frequency motifs across different interpreting outputs in pursuit of the sequential generation behaviors. It is found that the distribution, the length and certain position-dependent properties of the specific language sequences differ significantly across simultaneous interpreting and consecutive interpreting output. The features of frequency motifs manifest that both interpreting output is produced in the manner that abides by the least effort principle. The current research suggests that interpreting types can be differentiated through this type of language sequential unit and offers evidence for how the different task features mediate the sequential organization of interpreting output under different demand to achieve cognitive load minimization.

## Introduction

Interpreting is a particularly demanding language processing task for the cognitive system (Moser-Mercer, 2000; Christoffels et al., 2006; Pöchhacker, 2015; Dong, 2018; Liang et al., 2018). Such difficulties include the intensity and continuity of new speech input (Christoffels et al., 2006), the simultaneity of listening, retaining, comprehending the input and orally rendering the output, and the conflict and intervening effect of the concurrent activation of two languages (Gerver, 1976; Christoffels and De Groot, 2004; Christoffels et al., 2006). It is postulated that diversified cognitive constraints are at work in various interpreting types, mainly simultaneous interpreting (SI) and consecutive interpreting (CI). Though both interpreting modes require types of attention-sharing and overloading of working memory (Cowan, 1995; Gile, 2008), previous corpus- and treebank-based studies have demonstrated that SI and CI tax cognitive capacity differently and thus yield output of distinctive lexical and syntactic features (Liang et al., 2017; Lv and Liang, 2018).

Interpreting types have been explored and discussed in different theoretical models. As defined by Pöchhacker (b), SI is produced in synchrony with the interpreter’s perception and comprehension of the original utterance, with a processing-related time lag of a few seconds between the original utterance and interpretation. A majority of the SI models developed so far have attempted to track the interplay of the main operational tasks in one single step (Kirchhoff, 1976/2002; Lederer, 1981; Darò and Fabbro, 1994; Gile, 2009). Conversely, CI can be described as a two-stage process, that is, the source speech comprehension is followed by the re-expression in another language (Gile, 2009; Pöchhacker, 2011a). This mode of interpreting is performed in such cases where speakers prefer to finish a complete session before he “pauses for interpretation” (Pöchhacker, 2011a), such as in international press conferences. Faced with the need to render speeches lasting up to 20 min or more, interpreters may resort to note-taking to assist phonological memorization. In the framework of the Effort Models for instance, Gile (2009, 2016) outlines two separate stages in CI.

(1) **Comprehension phase: L + M + NP + C**
  L: Listening M: Short-term memory NP: Note Production C: Coordination
(2) **Reformulation phase: NR + SR + P + C**
  NR: Note Reading SR: Speech Reconstruction from Memory P: Production

By contrast, SI is modeled into a one-step process consisting of simultaneous efforts:

SIM=L+M+P+C

With the theoretical models repeatedly emphasizing the distinctive processes of SI and CI, empirical researches have rarely touched upon the differences between SI and CI output directly. Among the few is a debate on whether greater accuracy is achieved in CI or SI. Gile (2001), for instance, investigated how CI and SI interpreters cope with the potential problem triggers and found that CI interpreters were inferior in terms of overall accuracy. The opposite findings (Russel, 2002), however, claim that a higher level of accuracy is found for CI interpreters. As an initial effort in quantifying interpreting types, the results of the tree-bank based research (Liang et al., 2017) suggest different syntactic reformulation processes in SI and CI. In SI, the features of source language, including syntactic structures, have an essential impact on those of the output speech, and thus the mean dependency distance for the output speech is highly constrained by input. By contrast, CI formulates the target speech independently from the time course of the input, with fewer syntactic constraints from the source speech. Consequently, no such alignment of the mean dependency distance between the output and the input is found in CI.

The relative paucity of direct comparisons between SI and CI on the distinct processes renders it still an open question what exactly the different underlying mechanisms are in these two tasks. One possible reason is the lack of operational indicators, which leads to the rationale of the present study. The previous treebank-based study has demonstrated that SI and CI outputs differ in dependency distance (Liang et al., 2017), but the treebank is generated on the basis of dependency grammatical annotation and parsing, and thus is grammatically bound. The present study, on the other hand, employs a non-grammatically involved information by using frequency motif (F-motif) to address this issue.

Motif is a prototypical example of language sequence. As suggested, motif is a simple and machine-operable technique to determine and process linguistic sequential information, which proves to be a reliable approach to automatic text classification (Köhler, 2008). The idea of motif was recently transferred from musicology into linguistics by Köhler (2005, 2006, 2008), and today it enjoys an increasing interest (for reference, see Mačutek, 2009; Köhler, 2015; Mačutek and Mikros, 2015; Liu and Liang, 2017). As is emphasized above, motif is “a unit … which can give information about the sequential organization of a text… without relying on a specific linguistic approach or grammar” (Köhler, 2015). By utilizing “language in the line” features of texts, a motif is defined as *the longest continuous sequence of equal or increasing values representing a quantitative property of a linguistic unit (e.g., of morphs, words, or syntactic construction types).*

Accordingly, a F-motif can be constructed as “a continuous series of equal or increasing frequency values (Köhler, 2008). Each F-motif thus represents a series of words with non-decreasing frequencies in the texts, and the series can be employed to examine the sequential linguistic features of any text.

First and foremost, as a linear syntagmatic/sequential unit of word frequency, F-motif can visualize the local distribution of function words in the sentences (Köhler, 2008; Liu and Liang, 2017). As is known, function words are generally the most frequent elements in natural human languages and thus their sequential positions are strongly correlated with other word order phenomena (Greenberg, 1963; Dryer, 1992). This means that determining the relative order of function and content words might be a powerful cue to a large number of syntactic structures in a language (Gervain et al., 2013).

For instance, the frequency value of each token in the sentence On trade issue we have always maintained that trade disputes should be resolved through consultations from a certain corpus was determined based on the given file. The result is shown in Table 1 below.

**Table 1:** Frequency values of ‘On trade issue we have always maintained that trade disputes should be resolved through consultations.’

On	23
Trade	14
Issue	6
We	44
Have	29
Always	3
Maintained	1
That	70
Trade	14
Disputes	1
Should	8
Be	24
Resolved	1
Through	3
Consultations	3

Thus, the F-motifs of this sentence were generated according to the definition: (23) (14) (6-44) (29) (3) (1-70) (14) (1-8-24) (1-17) (3). There are six function words (i.e., articles, conjunction like “that,” prepositions like “on,” pronoun like “we,” and non-lexical verbs such as do, be and have) and nine content words (i.e., nouns like “trade,” “issue,” “disputes,” and “consultations,” lexical verbs like “maintain,” “should,” “resolved,” “adjectives,” adverbs like “always,” numerals and ordinals). It can be observed even in this short sentence that the frequencies of the content words (the highest is 14 and the mean is 6.25) are much lower than those of the function words (the lowest is 17 and the mean is 34.5). This difference can be even more illustrative when discussed in terms of word sequences, and herein F-motifs. Firstly, any features of the F-motif are equivalent to its counterparts of specific serial word sequences in the texts, and thus the distribution of F-motif is exactly the distribution of each serial word sequence in the given text. Secondly, it reveals how the sentence is truncated by the function words of higher frequency values, or in other words it shows the relative position of function and content words in a local context. Thirdly, since the last items of F-motif are likely function words, the length of F-motifs is also closely correlated with the local density of function words. Fourthly, the frequency values of each position in F-motifs of different-lengths can reflect the choice of content words in different relative position to local function words.

Moreover, when the motifs in language production are studied in a quantitative context, they are reflective of how people deal with the demand in the process of text (or speech) generation. A confirmation is that motifs display a lawful distributional behavior similar to other well-known linguistic units (Köhler and Naumann, 2008). According to the “principle of least effort” (Zipf, 1949), word frequency is a strong indicator of speakers’ tendency toward the minimization of production effort. That is, people tend to choose the most frequent words since the availability of a word is positively correlated with its frequency (Gernsbacher, 1994; Ferrer i Cancho and Sole, 2003). The maximally economical compromise between the competing needs of both the speaker and the hearer is argued to be the kind of reciprocal relationship between frequency and rank, to achieve easier production and better comprehension. Corresponding tests on the data of various motif types corroborate this hypothesis, showing a rank-frequency distribution of the sequences according to the Zipf-Mandelbrot (ZM) distribution (Beliankou et al., 2013). Hence, people have a preference for the more frequently used sequential units of language aside from word choices.

Furthermore, interrelations between length and frequency of sequence types are also expected to reveal certain properties of the sequential units and are constantly under investigation. According to synergetic linguistics, language systems present ‘self-organization’ and ‘self-regulation’ features in terms of the distribution of its linguistic units (Köhler, 2005). Accordingly, the length and frequency of sequence types are fitted with Menzerath-Altmann Law and others in analogy to known functional laws and hypotheses (Köhler, 2008; Liu and Liang, 2017). The data fitting demonstrates that people balance between the frequency and length of language sequences, which might be a result of the underlying features related to the length of motif.

So far, the empirical description of the statistics of motif sequences has been used for the comparison of authors (Biemann et al., 2016; Al Rozz and Menezes, 2018), texts (Chen, 2017), genres (Wang, 2017), languages (Chen and Liang, 2017; Jing and Liu, 2017; Mikros and Macutek, 2017), and for classification purposes (Köhler and Naumann, 2010; Liu and Liang, 2017). Given the correlation between word order and human cognitive functions (Dryer, 1992), the investigations into the language sequential units relative of function words distribution can provide a better illustration of the syntactic processing mechanisms attributing different interpreting styles and complement with the previous results based on grammatical annotations.

To sum up, the applicability of the regulation of motif in the basic linguistic level has been verified. However, previous studies generally use written materials as the subject of study, while the sequential units in spoken context were seldom explored. Since the spoken utterances are generally “extemporary” and produced one after another in sequences as opposed to the possible planning and revision in writing contexts, the sequential-related properties may provide us better insights into authentic spoken materials. Moreover, the synergetic linguistics argues that in the self-organizing language system, the order parameters mediating between the needs of the language users and the mechanisms of production and perception is dominated by the requirement to minimize the production effort and memorization effort (Köhler, 2005). Since interpreting is a cognitively demanding activity entailing both memory and production efforts, interpreters may seek to yield outputs with the least possible “effort.” This assumption has been born out in the previous tree-bank based research that shows a tendency toward dependency distance minimization (Liang et al., 2017) and the corpus-based research that points out the preference for words with simplified lexical features (Lv and Liang, 2018). In this vein, the present study investigates the language sequences of interpreting output from a quantitative perspective in the pursuit of uncovering the processing profiles of interpreters in different working modes. Given the spontaneous, demanding nature of interpreting, the analysis of the rarely-discussed position-dependent properties of motif, may yield meaningful results.

The present study will explore whether the language sequences in the output are also sensitive to different interpreting types. The following specific questions will be examined:

(1) Can the frequency distribution of language sequential units of frequency classify interpreting types?
(2) Can the length distribution of language sequential units of frequency classify interpreting types?
(3) What are the position-dependent properties of the language sequential units of frequency in SI and CI output?
(4) What are the psychological motivations underlying the varied distribution of language sequences in SI and CI output?

## Materials and Methods

### Materials

The current research intends to verify whether distinctive sequential patterns exist in the output across different modes of interpreting. To realize this goal, we built a self-built parallel corpus with transcribed real-world materials for two sub-corpora, namely, (1) a CI corpus consisting of the English interpretations and the source texts in Chinese of press conferences of the National People’s Congress from 2009 to 2016; (2) a SI corpus made up of 21 English interpretations and the Chinese source speech of keynote speeches recorded at the Boao Forum of Asia, Davos Forum from 2009 to 2016, as well as BRICs summits, sessions of the U.N. General Assembly, and China-ASEAN conferences during that time period. Across the parallel corpus, the source language is Chinese and the target language is English, and all interpretations were carried out from the mother tongue into the interpreters’ second language. In order to achieve a valid contrast between SI and CI, files of approximately 57,000 words were selected from each sub-corpus of English interpretations and their Chinese source texts are selected accordingly. Table 2 presents the summary for the corpora.

**Table 2:** Sizes of sub-corpora.

Sub-corpora	Chinese/English	No. of files	Running words in texts
SI	English	21	57199
	Chinese	21	80802
CI	English	8	57154
	Chinese	8	76314
Total		48	271469

### Methods

Given that the frequency value of words is particularly susceptible to text size, the sub-corpora were segmented to balance the text size. Thus, each output English sample file has approximately 4,000 tokens to ensure the validity of comparisons between sub-corpora. The segmentation was made without splitting a complete paragraph, and 28 equally-sized English files were obtained. The Chinese source texts were segmented in accordance with the English segmentation and 28 Chinese files were obtained. The frequency values of these 56 files of similar size were counted through Antconc, and the F-motifs were determined with respect to the frequency values of words in the given file. F-motifs of all the files in SI and CI were formed by Perl programs like the example given in the previous sector.

Then, the rank frequency distributions of F-motifs of both output groups were determined by ZM distribution, which is proven to be well-fitted with rank-frequency distribution in most cases and meaningful for investigation concerning motif (Beliankou et al., 2013; Wang, 2017). With respect to the operational dimension, this fitting process can be performed by Altmann Fitter (Altmann-Fitter, 2013) according to the following formula:

(1) $$\begin{array}{l} \text{P}(x)=\frac{{\left(b+x\right)}^{-a}}{F(n)},\\ {\text{F}}_{\text{(n)}}\text{, x}=1,2,3,\ldots ,\text{n}(a\in R,b>-1,n\in N) \end{array}$$

(2) $$F_{\left(n\right)}=\sum_{i=1}^{n}{\left(b+i\right)}^{-a}$$

**FIGURE 1:**
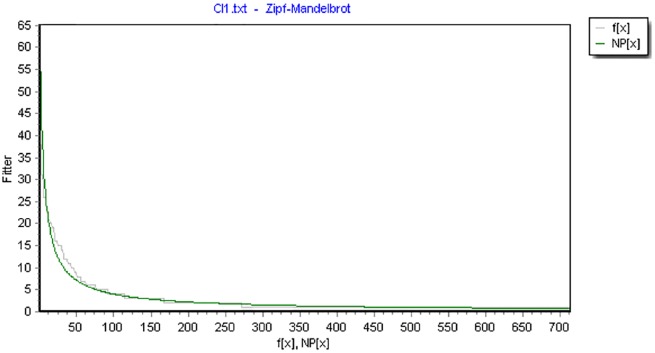
The rank frequency distribution of F-motifs modeled by ZM distribution (*df* = 574, *X*^2^= 99.9954, *R*^2^= 0.9496). (Note: the x-axis represents the rank number and the y-axis represents corresponding frequencies. F(x) is the observed frequency value and NP(x)is the predicted value.)

For instance, Figure 1 shows the model fitting of F-motifs of one text.

The two parameters in the function, i.e., *a, b* are obtained, and the fitting results across different interpreting types were examined. The parameter *a* determines the decay rate of distribution and the parameter *b* is a value that is strictly positive but no greater than 1. The parameters *a* is also “connected” to the changes in both low and high frequency words” (herein motifs), and is used as an indicator of linguistic change. The changes of *b* parameter, on the other hand, approximate changes of the class of low ranks words (Bentz et al., 2014; Koplenig, 2015). Correspondingly, the parameters of ZM models are used to classify languages according to the “grammatical fingerprint” (Bentz et al., 2014) and thus is also appropriate to identify the possible differences lying in the F-motifs of interpreting outputs. The lengths of F-motifs in SI and CI were also computed for further comparison. The length of F-motif was counted as the number of words it consisted of and the length distribution was tested to fit the Hyper-Pascal function, which is confirmed to be appropriate and frequently used for the length distribution in this line of studies (e.g., Köhler, 2005; Chen and Liang, 2017). The function is as follows:

(3) $$\begin{array}{l} \text{y}=\frac{\left(\begin{array}{c} k+x-1\\ x \end{array}\right)}{\left(\begin{array}{c} m+x-1\\ x \end{array}\right)}q^{x}p_{0},\;x=0,\;1,\;2,\ldots \\ {\text{P}}_{0}={{[}_{2}F_{1}(k,\;1;\text{ m};\;q)]}^{-1} \end{array}$$

The three parameters in the function, i.e., *m, k, q* were obtained, and the fitting results across different interpreting types were examined. The parameter *m* and *q* are reflective of the dependency of word frequency on length, with specific weights denoted and *k* indicating the number of components to be analyzed (Köhler, 2005). Additionally, the mean frequency value of words in each position of F-motifs was calculated. The statistics show that the vast majority (above 99%) of F-motifs produced in the present research were clustered around the length class from one to seven words. As a result, only the counts and frequency values in the position of one to seven were included in the calculation of the number and mean frequency in each position in the F-motifs.

## Results

Results are presented in three progressive aspects: (1) a classification of SI and CI output via a comparison of F-motif distribution parameters to fit the ZM models; (2) an investigation of the local distribution of function words in SI and CI by comparing the length of F-motifs; (3) identification of word choice preference in SI and CI by comparing the position-dependent frequencies.

### The Distribution of F-Motif in SI and CI

The rank frequency of F-motifs in SI and CI are fitted with ZM distribution and the parameters extracted from these models are further analyzed between SI and CI. Fitting the ZM distribution to the data of total F-motif tokens in the output yields excellent results. Models fit are all excellent according to *R*^2^ value shown in Table 3 (*R*^2^> 0.9). It must be remarked that the ZM distribution is one of the several models capturing the given data.

**Table 3:** Parameters of ZM model for F-motifs of SI and CI.

	Parameters							
Group	a	b	X^2^	P(X^2^)	df	C	*n*	*R*^2^
SI	0.9449	5.3162	1148.5143	0.0000	6469	0.0395	8225	0.9748
CI	0.8787	36.3978	562.2163	0.0000	6412	0.0544	8325	0.9446

**FIGURE 2:**
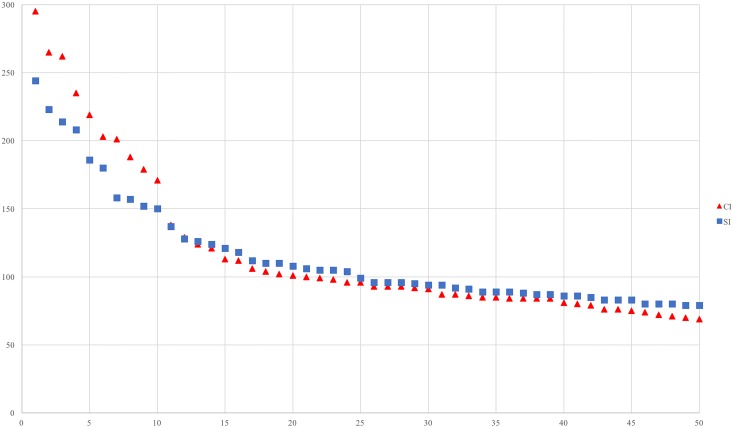
Rank frequency distribution of the highest 50 frequency F-motifs in SI and CI.

As can be seen in Table 3, the fittings are successful, which indicates that the collections of F-motifs of both interpreting types form a self-organizing system (Liu and Liang, 2017). And the distributions of the highest 50 frequency F-motifs of each group are illustrated in the bi-logarithmic graph in Figure 2.

The mean frequency of the highest 50 frequently occurred F-motifs is higher in CI (118.16) than SI (110.56), and that of the 20 most frequently occurred F-motifs is also higher in CI (168.4) than SI (151.42).

We then applied the Altmann-Fitter to all the 28 texts for analysis, and extracted the parameters for each file, which are listed in Supplementary Table 1.

**FIGURE 3:**
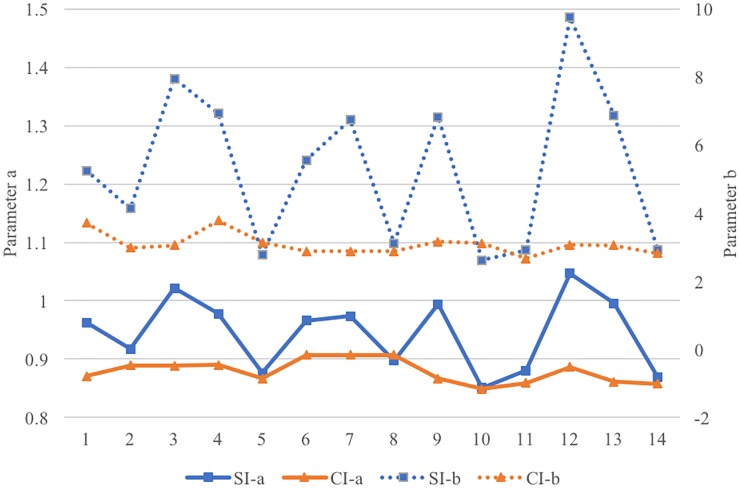
The values of parameter a and b in SI and CI. (The values of parameter a is shown in the left column of y-axis and the value of parameter b is shown in the right column.)

Independent sample *t*-tests on parameter *a* and parameter *b* across CI and SI are carried out respectively. The results show that parameters for the two groups are significantly different. Parameter *a* of SI (*M* = 0.945, *SD* = 0.062) is significantly higher than that of CI (*M* = 0.878, *SD* = 0.021), *t*_(26)_ = 3.778, *p* = 0.001, Cohen’s *d* = 1.447. Parameter *b* of SI (*M* = 5.388, *SD* = 1.47) is significantly higher than that of CI (*M* = 3.087, *SD* = 0.031), *t*_(26)_ = 5.714, *p* < 0.001, Cohen’s *d* = 2.213. Both parameters of SI show greater variances than those of CI, as is shown in Figure 3.

Since interpreting is a process mediating between source language and target language, the variance in the output might be attributed to the differences in source texts. In order to determine the possible reasons for the divergence, the rank frequency distribution of the F-motif of the source texts was applied to the ZM model, and the results are shown in Supplementary Table 2.

The F-motifs of Chinese input also present excellent fit with the ZM model, with a goodness of fit *R*^2^ generally higher than 0.968. Independent sample *t*-tests return no significant differences of parameters *a* or *b* between SI (*M* = 0.841, *SD* = 0.016 for a, *M* = 2.243, *SD* = 0.451 for b) and CI input (*M* = 0.840, *SD* = 0.013 for a, *M* = 2.31, *SD* = 0.414 for b) (*p* > 0.05).

To further test the possible effect of the input text on the output text in terms of the distribution of F-motifs, a zero-lagged Pearson correlation was calculated. The planned positive correlation was found only for SI group, parameter *a*: *R* = 0.678, *p* = 0.008, and parameter *b*: *R* = -0.605, *p* = 0.022, two-tailed. For SI, the distribution of the input F-motif explained a certain amount of the variances in the output F-motif, *F*_(1,12)_ = 10.203, *p* = 0.008, *R*^2^Adjusted = 0.414 for parameter *a* and *F*_(1,12)_ = 6.928, *p* = 0.022, *R*^2^Adjusted = 0.313 for parameter *b*. No such correlation is found for CI input and output.

Another factor of potential influence on the output of interpreting is the individual styles of interpreters (Van Besien and Meuleman, 2008). To examine whether individual difference contributes to variances in the distribution of F-motifs, we conducted a comparison of outputs produced by different interpreters in CI. In our collection, three interpreters are involved, who are all well-trained expert interpreters, working as commissioners of the Translation Department of China’s Ministry of Foreign Affairs. The distribution of the F-motif of their outputs shows no significant difference, Interpreter 1 (a = 8,796, b = 3.354), Interpreter 2 (a = 0.8775, b = 3.046) and Interpreter 3 (a = 0.8868, b = 3.023), *p* = 0.844 for parameter *a* and, *p* = 0.462 for parameter *b*. Hence, the individual interpreting style of various interpreters is ruled out as a factor leading to the differences between SI and CI in terms of F-motif distribution.

### The Length of F-Motif in SI and CI

Distinctive SI and CI output are determined with the distribution patterns. To further understand the effect of interpreting types on the distribution of function and content words in the target language, the length of F-motif in SI and CI are compared. Two approaches were performed: (1) the comparison of parameters of the fit models; (2) the comparison of the numbers of shorter and longer F-motifs.

**FIGURE 4:**
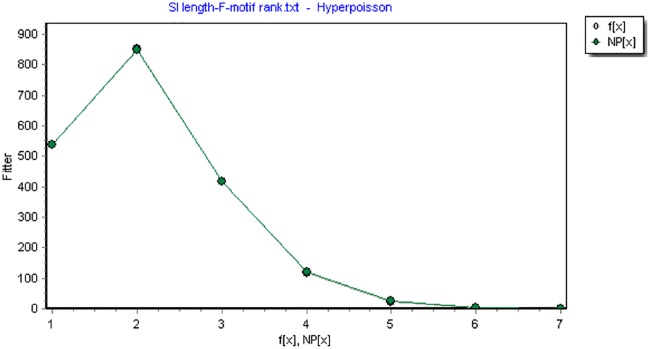
The length distribution of F-motifs modeled by Hyper-Pascal distribution (*df* = 3.0741, *X*^2^= 3.7291, *R*^2^= 0.9994). (Note: the x-axis represents the length and the y-axis represents corresponding frequencies. F(x) is the observed frequency value and NP(x) is the predicted value.)

The lengths of F-motifs in both interpreting types fit well with Hyper-Pascal distribution, with *R*^2^ generally over 0.99, as is shown in Figure 4 and Table 4.

**Table 4:** Parameters of Hyper-Pascal model in fitting to length distribution of F-motifs of SI and CI.

	Parameters							
Group	*k*	*m*	*q*	X^2^	P(X^2^)	df	C	*R*^2^
SI	1.0708	0.1833	0.2478	25.6751	0.1845	3.0000	0.0132	0.9921
CI	1.4574	0.2544	0.2290	28.9415	0.6634	3.0000	0.0148	0.9902

We then applied the Altmann-Fitter to all the 28 texts for analysis, and extracted the related information for further comparison (see in Supplementary Table 3). Independent sample *t*-tests results show significant differences of parameter *k, m, q* between SI and CI: parameter *k* (*M* = 1.100, *SD* = 0.28 for SI, *M* = 1.622, *SD* = 0.746 for CI, *t*_(26)_ = -2.454, *p* = 0.021, Cohen’s *d* = -0.926; parameter *m* (*M* = 0.184, *SD* = 0.043 for SI, *M* = 0.269, *SD* = 0.097 for CI, *t*_(26)_ = -2.976, *p* = 0.006, Cohen’s *d* = -1.133); and parameter *q* (*M* = 0.246, *SD* = 0.015 for SI, *M* = 0.225, *SD* = 0.027 for CI, *t*_(26)_ = 2.555, *p* = 0.017, Cohen’s *d* = 0.962).

Again, we checked the length distribution of F-motifs of the input texts in Chinese and independent sample *t*-test was conducted to determine the possible difference in parameters (see in Supplementary Table 4). No significant differences are found in parameter *k* of SI (*M* = 0.91, *SD* = 0.41) and CI (*M* = 1.04, *SD* = 0.0428), *p* = 0.403, parameter *m* of SI (*M* = 0.213, *SD* = 0.093) and CI (*M* = 0.217, *SD* = 0.081), *p* = 0.911 and parameter *q* of SI (*M* = 0.318, *SD* = 0.029) and CI (*M* = 0.298, *SD* = 0.026), *p* = 0.056. Thus, source texts can be rule out as a factor that contributes to the distinct length distributions of F-motif in SI and CI.

The results of ANOVA test also rule out the possible effect of interpreting style of the three interpreters on the length of F-motif in the output, *F*_(2,11)_ = 0.856, *p* = 0.451 for parameter *k*; *F*_(2,11)_ = 0.259, *p* = 0.777 for parameter *m*; *F*_(2,11)_ = 0.226, *p* = 0.451 for parameter *q*.

Hence, neither the source text nor the interpreting style of varied interpreters underlies the variances in the length differences of F-motif in SI and CI output.

**FIGURE 5:**
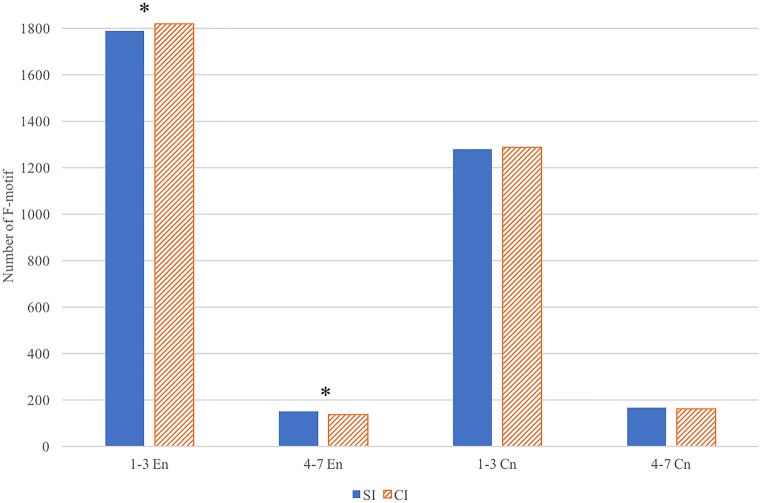
The number of shorter (1–3 words) F-motifs and longer (4–7 words) F-motifs in SI and CI output (^∗^ indicates to where significant difference is detected).

Next, a comparison of the total number of shorter (1, 2, and 3 words) and longer (4–7 words) F-motifs between SI and CI was conducted with an independent sample *t*-test. It is found that the shorter F-motifs of CI (*M* = 1819.213, *SD* = 43.349) is significantly larger in number than that of SI (*M* = 1789.500, *SD* = 26.924), *t*_(26)_ = -2.179, *p* = 0.039, Cohen’s *d* = -0.823. On the contrary, there are more longer F-motifs in SI (*M* = 149.286, *SD* = 19.277) than in CI (*M* = 136.929, *SD* = 8.946), *t*_(26)_ = 2.176, *p* = 0.039, Cohen’s *d* = 0.822. The patterns are shown in Figure 5. It should be noted that there is no significant difference in the total number of F-motifs in SI (*M* = 1959.929, *SD* = 59.486) and CI (*M* = 1959.143, *SD* = 33.240) output, *t*_(26)_ = 0.043, *p* = 0.966. Moreover, the independent sample *t*-tests on the proportion of F-motifs in the input texts return no significant differences between SI and CI in shorter F-motifs (*M* = 1278.210, *SD* = 154.649 for SI, *M* = 1287.930, *SD* = 122.986 for CI, *t*_(26)_ = -0.184, *p* = 0.855), or longer F-motif (*M* = 167.140, *SD* = 16.489 for SI, *M* = 153.93, *SD* = 22.113 for CI), *t*_(26)_ = 1.677, *p* = 0.105.

### Position-Dependent Properties F-Motif of Interpreting Types

In the previous section, it is found that both the distribution and length of F-motif in interpreting output differ across interpreting groups. More information regarding the function and content word choices can be attained if we re-assess the data from a perspective of the position-related information of the F-motif.

**FIGURE 6:**
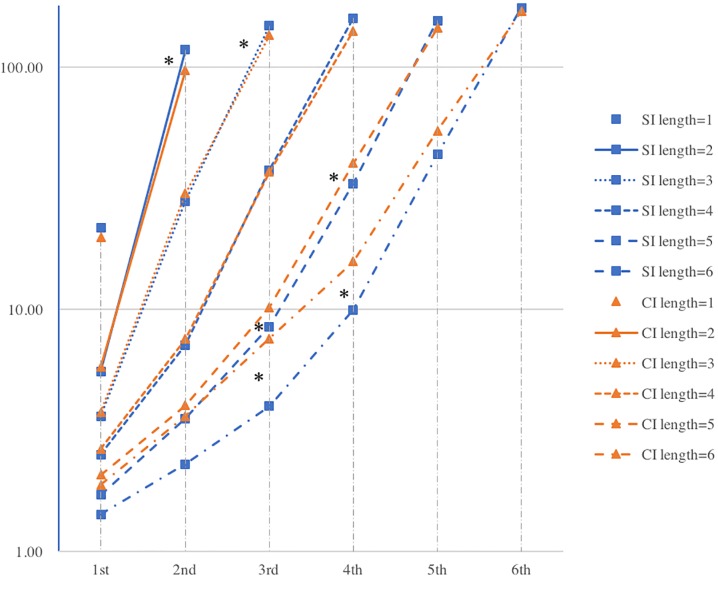
Logarithmic mean frequency of words in each position in F-motif of SI and CI (^∗^ indicates to where significant difference is detected).

A (reversed) interrelation between the length and frequency of linguistic units/sequences has been confirmed (Köhler, 2008; Köhler and Naumann, 2010) whereas the property of each position in the sequence is rarely discussed. Thus, the log-transformed mean frequencies in each position of F-motifs of SI and CI are extracted and are illustrated in Figure 6.

The mean frequency values in each position in F-motif across groups generally appear the same patterns: (1) the mean frequencies of the last position in each F-motif length are generally higher in SI than in CI; (2) the mean frequency values of each position except the last position in each length of F-motif is generally higher in CI than in SI.

More specifically, in shorter F-motifs, the last positions present a significantly higher mean frequency in SI than CI while no significant differences are detected in other positions. For instance, the last positions of two-word (L2P2) and three-word F-motifs (L3P3) show significant differences. In L2P2, SI (*M* = 118.399, *SD* = 10.004) is significantly higher than that in CI (*M* = 97.272, *SD* = 11.304), *t*_(26)_ = 5.236, *p* < 0.001, Cohen’s *d* = 1.979; in L3P3, frequency value of SI (*M* = 148.674, *SD* = 12.270) is significantly higher than that of CI (*M* = 135.880, *SD* = 22.590), *t*_(26)_ = 2.863, *p* = 0.02, Cohen’s *d* = 0.704. On the contrary, in longer F-motifs, there is no significant difference in the last position whereas the mean frequency in other positions are significantly higher in CI than in SI. For instance, significant variances are identified in position three and four in five-word (L5F3, L5F4) and six-word motif (L6F3, L6F4). In L5F3, the frequency value of CI (*M* = 10.194, *SD* = 2.141) is significantly higher than that in SI (*M* = 8.494, *SD* = 1.961), *t*_(26)_ = -2.191, *p* = 0.038, Cohen’s *d* = 0.828; in L5F4, the frequency value of CI (*M* = 40.248, *SD* = 6.853) is significantly higher than that in SI (*M* = 33.160, *SD* = 6.641), *t*_(26)_ = -1.991, *p* = 0.044, Cohen’s *d* = 1.05; in L6F3, the frequency value of CI (*M* = 7.57, *SD* = 5.309) is significantly higher than that in SI (*M* = 3.990, *SD* = 1.908), *t*_(26)_ = -2.376, *p* = 0.025, Cohen’s *d* = 0.897; in L6F4, the frequency value of CI (*M* = 15.79, *SD* = 6.091) is significantly higher than that in SI (*M* = 9.95, *SD* = 3.459), *t*_(26)_ = 2.057, *p* = 0.045, Cohen’s *d* = 1.179.

Furthermore, the frequency values of different position point to different words or word classes in the text. On the one hand, it is found that the content words with the highest frequency value in all CI text is “China,” and its mean frequency is 42.21. The most frequently used content words in SI are “China,” “development” and “economic,” the mean frequency of which are 59.5. Thus, the words in the last position of F-motif of all lengths (except one-word F-motif) are very likely function words. On the other hand, the words in the third and fourth position of longer F-motifs are mostly content words according to the frequency value.

## Discussion

The current research is the very first effort investigating the different linguistic features of SI and CI output by employing a linguistic sequence visualizing the local distribution of function words without relying on grammatical parses. This study complements previous treebank-based studies by quantitatively examining the non-grammatically-bound language sequences in different interpreting outputs. It is further confirmed that the output of different interpreting types, differs not only in dependency parsed information, but in the local, sequential distribution of function words. Given that the distribution of F-motif abides by the principle of least effort, the current findings highlight the different mechanisms in SI and CI in realizing production and memory effort minimization.

Our results indicate that the output texts of SI and CI entail F-motifs of different distribution, lengths and position-dependent frequencies, regardless of the differences in text size, input texts or the interpreting style of individual interpreters. To be specific, it is found out that: firstly, only the distribution of SI output F-motifs is significantly correlated with that of input; secondly, CI generates more short F-motifs (one-to-three words motifs) while SI produces more long F-motifs (four-to-seven word motifs); and thirdly, the mean frequencies of content words in the same position of the long F-motif in CI are higher those in SI.

### The Distribution of F-Motif Across Interpreting Types

The present study first compares the ZM parameters fit by the F-motif in SI and CI ouput. Though they both fit the same ZM model, significant differences are found even when the influence of input text and individual style of interpreters are excluded. The different patterns demonstrate that SI and CI outputs are two distinctive inter-languages and that different operational mechanisms are involved in the processes.

In addition, it is indicated in the correlation tests that only SI output is significantly affected by the input in terms of the frequency of these language sequences. This result is a manifestation that the sequential organization of the output in SI is closely constrained by the input whereas CI reformulation is more independent. It corroborates the findings in previous studies comparing the dependency distance of SI and CI output (Liang et al., 2017). They found that the dependency distance of SI output approximates that of the source language, but the dependency distance of CI output is significantly shorter. They argue that SI interpreters produce syntactic structures closely in line with those of the source language due to the concurrent processes of source speech comprehension and target speech production; by contrast, as speech comprehension and production in CI are temporally separated, the interpreters are relatively “self-paced.” Thus, CI interpreters would prefer to generate simpler syntactic structures to lessen the burden on processing, and thus dependency distance minimization occurs.

The results of the present study favor this proposition. On the one hand, the input F-motif has an essential impact on the output F-motif of SI but no such correlation is found for CI. In the quantitative context, F-motif distribution in the input text can explain about 40% of the variances in that of the output of SI. In the local context, it means that the sequential frequency values of the output in SI is synchronized with those of the input. However, no significant correlation is found for the distribution of F-motifs between the input and the output of CI. Thus, we speculate that SI is produced closely in line with the input text, thus the linear sequences of word frequency of the output are distributed in alignment with those of the input.

On the other hand, ZM parameters of F-motifs in SI output vary a lot while those of CI are limited to a small range. Since the parameters of the input F-motif of both interpreting types fluctuate, it is assumed that the clustering of the parameters of the F-motifs in CI output is attributed to the mediation effect in the interpreting process. In other words, instead of retaining the diverse sequential orders of the source text, CI interpreters may tend to employ more frequently used structures or sequences and thus yield F-motifs bearing more regular and consistent distributions. This assumption fits squarely into the fact that the F-motifs of CI output show a greater central tendency as the mean frequency of the most frequently occurred F-motifs (top 50) is higher in CI than SI, and the standard deviation is larger in SI than CI. In sum, to lessen the processing difficulties, SI interpreters tend to follow the sequences of the input whereas CI interpreters not only adopt structures of less complexity but also employ more frequently used language sequences.

### The Length of F-Motif Across Interpreting Types

The results for fitting the length distributions of SI and CI F-motif to models corroborate with the length distribution of length-motifs of written texts, as both fit well with the Hyper-Pascal model (Liu and Liang, 2017). However, compared to the motifs of written texts, the lengths of F-motif in the present study are generally shorter and 99% of the F-motifs cluster at the length values of 1, 2, and 3. Furthermore, shorter F-motifs account for a larger proportion in CI output than in SI output, and the difference is attributed to the interpreting process rather than the variances in the input text or corpora size.

It is postulated that the distinctive types of text (written vs. spoken) contribute to the different length distributions of motifs. Drawing on evidence from spoken language corpora and multiple languages, Green (2017) discovers that the average length of recurrent word sequences aligns with the WM capacity estimate of Cowan (2001) and the recurrent phrases of or five or more words are less than 1% of all tokens. It has also been demonstrated experimentally that phrase frequency alone is cognitively retained and has processing advantages (Sosa and MacFarlane, 2002; Arnon and Snider, 2010). They thus claim that the requirement of minimization in producing effort appears to have a more apparent manifestation in the spoken language than written language. In the same vein, we assume that the length distribution of F-motif captures the processing load during interpreting.

F-motif, a sequential unit consisting of words of non-decreasing frequency, can be regarded as word bundles segmented by high-frequency words. As mentioned above, function words and content words are dispersed asymmetrically on a continuum of the frequency value in each text. Most of the function words are of high-frequency and they are either the one-word F-motifs or the words in the last position of F-motif sequences. The longer the F-motif is, the more content words are in the sequence. Thus, shorter F-motifs can be indirectly linked with a dense distribution of function words. It is concluded in consequence that function words are more densely distributed in CI than SI output, which is possibly due to the different mechanisms of producing sequences during the two interpreting types.

It is generally believed that SI interpreters, constrained by the temporal pressure, handle the source speech in piecemeal (Padilla et al., 2005; Morales et al., 2015). Thus, the rich variety of texture signals has to be relied upon the most tangible point of reference, i.e., the content words. To avoid the possible cognitive resources saturation, the information retained in the focus of attention for processing should be kept as small in amount as possible. Hence, SI interpreters usually produce the output without much altering the sequence of source text elements, avoiding the increased pressure which would have been entailed by reordering the parts (Hatim and Mason, 2002; Lin et al., 2018). In this way, the chunks of information can be relieved from the focus of attention immediately after they are formulated in the target language (Liang et al., 2017; Mizuno, 2017).

Conversely, CI interpreters receive speakers’ uninterrupted utterances in portions of at least a few sentences. Though interpreters in this working mode are not taxed much attention from the simultaneous presentation of input and output speech, more time is required in taking notes but only part of the information can be taken down. Thus, it generates an added pressure and extra load on working memory (Gile, 2009). Meanwhile, CI interpreters are more self-paced in the reformulation phase, thus they can choose to negotiate meaning in a less demanding manner. Hatim and Mason (2002) stressed the prominence of structure in CI. It is claimed that in CI, the texture- and context-related information is too detailed and can only be retained in a most short-lived manner. Thus, to achieve effective storage, more structure-related information is used for better retaining and processing. An effective CI output thus exhibits an outline of the way a text is structured while some texture- and context-related information may not be retained as detailed as in the input (Hatim and Mason, 2002). Intuitively, function words, due to their high frequency, act as anchor points with respect to which the structural roles and sequential positions of other constituents can be encoded and remembered (Gervain et al., 2013). Consequently, CI interpreters may tend to retain more structure-related information and generate output accordingly to lessen the burden on working memory. This preference is consistent with the universal preference for Least Effort (Zipf, 1949). In other words, the high cognitive load in the reformulation phase in CI forces interpreters to generate output with densely distributed structure-related, function words, which accounts for the larger proportion of shorter F-motifs in CI.

### The Position-Dependent Properties in F-Motif Across Interpreting Types

In the present study, two notable differences of position-dependent frequencies of F-motifs are detected: (1) the mean frequencies of the last-position words in shorter F-motifs are higher in SI than in CI; (2) the mean frequencies of the third and fourth positions in longer F-motifs are higher in CI than in SI.

The frequencies of L2P2 and L3P3 are significantly higher in SI than CI F-motifs. An exhaustive search for words falling in the frequency range of L2P2 and L3P3 finds out that they belong to the same top-frequency function words (in, to, of, and, the) in both groups. Thus, there is no practical difference whatsoever between SI and CI in terms of content word or function word choices in shorter F-motifs.

Statistically, only the mean frequencies of the third and fourth positions of longer F-motifs (L5P3, L5P4, L6P3, and L6P4) are significantly higher in CI than SI. A further check indicates that the frequency values in these positions mainly point to content words. No significant differences between SI and CI were found in the aspect of function words. In other words, the function word usage in longer F-motifs does not differ between SI and CI. However, the output of the two interpreting types varies in content word choices in longer F-motif. As is emphasized above, the length of F-motif is indirectly related to the distribution of content and function words. Longer F-motif consists of more content words and one function word, where content words are more densely distributed. As a result, the position dependent differences signify that CI interpreters tend to use more frequently used words than in SI when function words are not locally accessible. It has been argued in the previous section that interpreters tend to rely on structural information to memorize input messages and generate more function words in the output sequences to alleviate working memory burden. When there is less structure-related information in the sequence, more pressure is imposed on the CI interpreter, who might resort to high-frequency, polysemous content words to lessen the production load (Lv and Liang, 2018). SI interpreters, on the other hand, focus more on the textual clues and are not so much influenced by the lack of grammatical words (Hatim and Mason, 2002).

To recap, we assume that two processing approaches underlie the differences between SI and CI output in terms of the sequential organizations. For SI, the simultaneity nature poses high demand on the coordination between input and output, guiding the interpreters’ efforts to retain the textual sequences of the input text. Conversely, CI interpreters store and reformulate the messages effectively via structure related information to lessen the memory and processing load. Thus, they tend to produce more frequently used sequences, where function words are more densely distributed. Or otherwise, CI interpreters adopt frequently used content words if less function words are accessible for scaffolding.

### The Application and Nature of F-Motif

Though the sequential linguistic units of motif have been introduced into the linguistic world for a short period of time, its application into linguistic research is promising.

Previous studies using different types of motifs have proved its usage in text, genre and language types classification. However, using F-motif in investigating interpreting uncovers its reflection of human cognitive constraints in producing language sequences. Essentially, types of attention-sharing and overloading of working memory are generally postulated to be the cognitive underpinnings of interpreting (Cowan, 1995; Gile, 2008). As different interpreting types have been modeled into varied cognitive procedures, the sequential production mechanism can be expected to show differences.

In the present study, the usage of F-motif is extended to quantitatively investigate the local distribution of function words and the sequential order of high and low frequency words in a given text. The sequential and distributional information can, to some extent, reflect the word choice and the sequential production mechanism of language, particularly spoken language. More importantly, it is shown that F-motif can be used to mirror the different types of cognitive demand involved in different tasks. Firstly, F-motif of interpreting can be modeled with ZM distribution model and its correlation test results with the input texts reflect whether the linear sequences of word frequency of the output are distributed in alignment with or independent of those of the input. Secondly, the length of F-motifs reflects the density of function words in word sequences, and thus mirrors the different mechanisms of producing sequences during the two interpreting types to minimize the storage and producing effort. For example, to alleviate storage burden, CI interpreters can rely more on structure-related information and thus generate the output with more densely distributed function words, which leads to more short F-motifs. Thirdly, in certain positions of long F-motifs, different word choices are also evidenced in these two interpreting types. It is noticed that CI interpreters tend to use more frequently used words than in SI when structural information is not locally accessible. In other words, the position-dependent frequency of F-motifs offers detailed explanation for word choices in sequential language production.

As suggested in a recent commentary, the quantitative studies on interpreting tasks and their underlying cognitive mechanisms under different circumstances serve as an arena for the integration of approaches to the investigation into language foundations and human cognitions (Liang et al., 2018). Language can coevolve with memory capacity, and the limited memory capacity also drives language transition. Studies on interpreting illustrate how language shifts under great cognitive load. The behavioral and neurological studies in this line suggest that interpreting training enhances and coevolves with domain-general cognitive functions (Van De Putte et al., 2018). The quantitative studies on interpreting types can shed light on an integrated effect of socio-cultural environment and domain-general abilities on language production. These works underline the view that language is shaped by cognitive constraints and socio-cultural environment. This also gives us good reasons to believe that interpreting serves as an appropriate subject of research on the foundation of language use and machine translation.

## Conclusion

The current research investigates the distribution, length and the position-dependent properties of a language sequential unit, F-motif, in SI and CI outputs. It is found that the distribution and the lengths of F-motifs differ significantly across SI and CI output. The mean frequencies of the content words in some positions of the longer F-motifs vary between SI and CI, which confirms the requirement of minimum producing and memory effort in interpreting process. The different sequence-related features of SI and CI output are the results of varied cognitive constraints involved in the interpreting processes and the correspondent coping mechanism of interpreters.

The present study may offer a novel method to differentiate different interpreting types and to quantify the differences in a reasonable way. Such a quantification can be viewed as an indicator of how far the real-world SI and CI output differs. Moreover, the sequential delivery of expert interpreters sets an example for novel interpreters, who should be trained specifically for each interpreting type. The length and position-dependent frequencies can be related to specific structural properties of interpreting types, which may very likely offer insights into the development of artificial intelligence in interpreting tasks. Other basic linguistic properties can be further investigated with this approach to better understanding the sequential processing in interpreting.

## Author Contributions

JL and QL conceived and designed the experiments. JL, QL, and YL performed the experiments, collected the data, and performed the data analyses. All authors contributed to the interpretation of results and the writing of the manuscript and approved the final version of the manuscript for submission.

## Conflict of Interest Statement

The authors declare that the research was conducted in the absence of any commercial or financial relationships that could be construed as a potential conflict of interest.
