# Supplementary material for: Quantifying Interpreting Types: Language Sequence Mirrors Cognitive Load Minimization in Interpreting Tasks
Source: Front Psychol. 2019 Feb 18;10:285. doi: 10.3389/fpsyg.2019.00285 (PMC6387939; doi:10.3389/fpsyg.2019.00285)
Supplement: Supplementary file 1 [file Table_1.DOCX]

Supplementary Table 1. Parameters of ZM model for F-motifs of SI and CI

| group | ID | a | b | X^2^ | P(X^2^) | DF | C | R^2^ |
| --- | --- | --- | --- | --- | --- | --- | --- | --- |
| SI | 1 | 0.9623 | 5.2554 | 63.6741 | 1 | 563 | 0.0320 | 0.9728 |
|  | 2 | 0.9175 | 4.1399 | 52.8459 | 1 | 571 | 0.0272 | 0.9793 |
|  | 3 | 1.0217 | 7.9571 | 70.8346 | 1 | 551 | 0.0365 | 0.9832 |
|  | 4 | 0.9780 | 6.9366 | 44.0202 | 1 | 553 | 0.0227 | 0.9853 |
|  | 5 | 0.8764 | 4.7824 | 57.7414 | 1 | 681 | 0.0299 | 0.9632 |
|  | 6 | 0.9661 | 5.5620 | 50.1717 | 1 | 544 | 0.0264 | 0.9888 |
|  | 7 | 0.9735 | 6.7564 | 52.2781 | 1 | 557 | 0.0273 | 0.9883 |
|  | 8 | 0.8974 | 3.1162 | 85.8430 | 1 | 580 | 0.0434 | 0.9192 |
|  | 9 | 0.9942 | 6.8309 | 46.3819 | 1 | 535 | 0.0244 | 0.9898 |
|  | 10 | 0.8509 | 3.6191 | 50.8968 | 1 | 610 | 0.0260 | 0.9716 |
|  | 11 | 0.8803 | 3.9126 | 60.3908 | 1 | 571 | 0.0309 | 0.9417 |
|  | 12 | 1.0468 | 4.7669 | 50.2667 | 1 | 534 | 0.0260 | 0.9877 |
|  | 13 | 0.9956 | 6.8804 | 73.4324 | 1 | 570 | 0.0363 | 0.9757 |
|  | 14 | 0.8685 | 4.9110 | 56.9653 | 1 | 638 | 0.0268 | 0.9658 |
| CI | 1 | 0.8703 | 3.7151 | 78.3534 | 1 | 607 | 0.0389 | 0.9208 |
|  | 2 | 0.8889 | 2.9924 | 62.9127 | 1 | 548 | 0.0323 | 0.9835 |
|  | 3 | 0.8883 | 3.0578 | 92.0208 | 1 | 589 | 0.0473 | 0.9034 |
|  | 4 | 0.8895 | 3.7815 | 102.6205 | 1 | 591 | 0.0526 | 0.8801 |
|  | 5 | 0.8666 | 3.1341 | 55.0165 | 1 | 599 | 0.0282 | 0.9633 |
|  | 6 | 0.9072 | 2.8795 | 88.6352 | 1 | 572 | 0.0442 | 0.9102 |
|  | 7 | 0.9072 | 2.8795 | 88.6352 | 1 | 572 | 0.0442 | 0.9102 |
|  | 8 | 0.9072 | 2.8795 | 88.6352 | 1 | 572 | 0.0442 | 0.9102 |
|  | 9 | 0.8664 | 3.1670 | 77.0513 | 1 | 615 | 0.0395 | 0.9479 |
|  | 10 | 0.8487 | 3.1103 | 42.6686 | 1 | 608 | 0.0225 | 0.9918 |
|  | 11 | 0.8587 | 2.6637 | 62.9023 | 1 | 614 | 0.0317 | 0.9627 |
|  | 12 | 0.8862 | 3.0739 | 72.5893 | 1 | 583 | 0.0370 | 0.9402 |
|  | 13 | 0.8610 | 3.0585 | 69.309 | 1 | 623 | 0.0351 | 0.9577 |
|  | 14 | 0.8573 | 2.8251 | 75.9449 | 1 | 614 | 0.0392 | 0.9464 |
