# Supplementary material for: Quantifying Interpreting Types: Language Sequence Mirrors Cognitive Load Minimization in Interpreting Tasks
Source: Front Psychol. 2019 Feb 18;10:285. doi: 10.3389/fpsyg.2019.00285 (PMC6387939; doi:10.3389/fpsyg.2019.00285)
Supplement: Supplementary file 2 [file Table_2.DOCX]

Supplementary Table 2. The parameters of ZM model for the rank-frequency distribution of the source texts of SI and CI.

| group | ID | a | b | R^2^ |
| --- | --- | --- | --- | --- |
| SI | 1 | 0.8533 | 2.1807 | 0.9742 |
|  | 2 | 0.8318 | 2.0726 | 0.9684 |
|  | 3 | 0.8514 | 1.9482 | 0.9832 |
|  | 4 | 0.866 | 1.6742 | 0.989 |
|  | 5 | 0.8416 | 2.1741 | 0.9902 |
|  | 6 | 0.8612 | 1.9285 | 0.9869 |
|  | 7 | 0.8591 | 1.9728 | 0.9926 |
|  | 8 | 0.8141 | 2.5741 | 0.9871 |
|  | 9 | 0.8424 | 2.0601 | 0.9848 |
|  | 10 | 0.8201 | 2.4433 | 0.9788 |
|  | 11 | 0.8247 | 3.4255 | 0.9723 |
|  | 12 | 0.8474 | 2.3845 | 0.9788 |
|  | 13 | 0.8355 | 2.3619 | 0.9734 |
|  | 14 | 0.8249 | 2.2064 | 0.9719 |
| CI | 1 | 0.8374 | 2.1979 | 0.9868 |
|  | 2 | 0.8427 | 2.2472 | 0.9818 |
|  | 3 | 0.8442 | 2.1996 | 0.9811 |
|  | 4 | 0.8467 | 2.3903 | 0.9719 |
|  | 5 | 0.845 | 2.1895 | 0.9828 |
|  | 6 | 0.8517 | 2.4685 | 0.9868 |
|  | 7 | 0.8444 | 2.2594 | 0.9752 |
|  | 8 | 0.8248 | 1.9983 | 0.969 |
|  | 9 | 0.8347 | 2.0632 | 0.9915 |
|  | 10 | 0.8574 | 3.6488 | 0.9887 |
|  | 11 | 0.8459 | 2.2407 | 0.9817 |
|  | 12 | 0.8497 | 1.9822 | 0.9802 |
|  | 13 | 0.8282 | 2.0427 | 0.9815 |
|  | 14 | 0.8055 | 2.4034 | 0.9821 |
