# Supplementary material for: Quantifying Interpreting Types: Language Sequence Mirrors Cognitive Load Minimization in Interpreting Tasks
Source: Front Psychol. 2019 Feb 18;10:285. doi: 10.3389/fpsyg.2019.00285 (PMC6387939; doi:10.3389/fpsyg.2019.00285)
Supplement: Supplementary file 3 [file Table_3.DOCX]

Supplementary Table 3. The parameters of Hyper-Pascal for the length distribution of F-motif in SI and CI output.

| group | ID | k | m | q | R^2^ |
| --- | --- | --- | --- | --- | --- |
| SI | 1 | 0.9403 | 0.1557 | 0.223 | 0.9931 |
|  | 2 | 0.9139 | 0.1624 | 0.2543 | 0.9934 |
|  | 3 | 0.9028 | 0.1577 | 0.2595 | 0.9947 |
|  | 4 | 0.9028 | 0.1577 | 0.2595 | 0.9947 |
|  | 5 | 1.1192 | 0.1731 | 0.2347 | 0.9882 |
|  | 6 | 1.4404 | 0.2371 | 0.2378 | 0.9896 |
|  | 7 | 0.7389 | 0.118 | 0.2682 | 0.9939 |
|  | 8 | 0.7971 | 0.1668 | 0.2692 | 0.9963 |
|  | 9 | 1.1039 | 0.1673 | 0.2502 | 0.9898 |
|  | 10 | 1.2871 | 0.2222 | 0.2451 | 0.9907 |
|  | 11 | 1.0619 | 0.1855 | 0.2461 | 0.9931 |
|  | 12 | 1.0334 | 0.1623 | 0.2458 | 0.9919 |
|  | 13 | 1.422 | 0.2436 | 0.2357 | 0.9902 |
|  | 14 | 1.7302 | 0.2729 | 0.2216 | 0.9875 |
| CI | 1 | 0.7888 | 0.1361 | 0.2484 | 0.9943 |
|  | 2 | 1.4924 | 0.2547 | 0.2291 | 0.989 |
|  | 3 | 2.0123 | 0.333 | 0.2072 | 0.9868 |
|  | 4 | 3.5213 | 0.4995 | 0.1658 | 0.978 |
|  | 5 | 2.6695 | 0.4097 | 0.1876 | 0.9843 |
|  | 6 | 1.1043 | 0.2056 | 0.238 | 0.9934 |
|  | 7 | 1.9655 | 0.2956 | 0.2056 | 0.9863 |
|  | 8 | 1.3896 | 0.2381 | 0.2247 | 0.9886 |
|  | 9 | 1.4868 | 0.2437 | 0.2262 | 0.9884 |
|  | 10 | 1.2038 | 0.2286 | 0.2606 | 0.9936 |
|  | 11 | 0.8974 | 0.1598 | 0.2522 | 0.9939 |
|  | 12 | 1.1681 | 0.2041 | 0.2381 | 0.993 |
|  | 13 | 1.1366 | 0.2338 | 0.2537 | 0.9945 |
|  | 14 | 1.8705 | 0.3255 | 0.2199 | 0.9896 |
