# Supplementary material for: Quantifying Interpreting Types: Language Sequence Mirrors Cognitive Load Minimization in Interpreting Tasks
Source: Front Psychol. 2019 Feb 18;10:285. doi: 10.3389/fpsyg.2019.00285 (PMC6387939; doi:10.3389/fpsyg.2019.00285)
Supplement: Supplementary file 4 [file Table_4.DOCX]

Supplementary Table 4. The parameters of Hyper-Pascal for the length distribution of F-motif in the source texts of SI and CI.

| group | ID | k | m | q | R^2^ |
| --- | --- | --- | --- | --- | --- |
| SI | 1 | 0.6216 | 0.1526 | 0.3296 | 0.9983 |
|  | 2 | 1.1052 | 0.2632 | 0.3139 | 0.9954 |
|  | 3 | 0.5834 | 0.1549 | 0.344 | 0.9977 |
|  | 4 | 1.0912 | 0.2525 | 0.3008 | 0.996 |
|  | 5 | 0.5401 | 0.1363 | 0.3186 | 0.9983 |
|  | 6 | 0.5499 | 0.1343 | 0.3263 | 0.9979 |
|  | 7 | 0.6457 | 0.1609 | 0.3322 | 0.9979 |
|  | 8 | 1.5178 | 0.3634 | 0.2864 | 0.9935 |
|  | 9 | 0.8957 | 0.1923 | 0.3159 | 0.9959 |
|  | 10 | 0.3383 | 0.0851 | 0.3716 | 0.9991 |
|  | 11 | 1.0947 | 0.2636 | 0.318 | 0.9961 |
|  | 12 | 1.3311 | 0.2834 | 0.28 | 0.991 |
|  | 13 | 1.7146 | 0.3994 | 0.2693 | 0.9925 |
|  | 14 | 0.7146 | 0.1448 | 0.3102 | 0.9951 |
| CI | 1 | 1.4135 | 0.2619 | 0.2534 | 0.9915 |
|  | 2 | 0.6669 | 0.1471 | 0.3104 | 0.9975 |
|  | 3 | 1.0075 | 0.2336 | 0.3043 | 0.9956 |
|  | 4 | 0.7591 | 0.1522 | 0.2896 | 0.9958 |
|  | 5 | 0.7562 | 0.1725 | 0.3046 | 0.9975 |
|  | 6 | 0.5704 | 0.121 | 0.3139 | 0.9974 |
|  | 7 | 0.9956 | 0.2012 | 0.286 | 0.996 |
|  | 8 | 1.825 | 0.3103 | 0.2395 | 0.9895 |
|  | 9 | 0.5524 | 0.1169 | 0.3224 | 0.9971 |
|  | 10 | 1.5919 | 0.3508 | 0.2663 | 0.9922 |
|  | 11 | 1.1297 | 0.2547 | 0.2859 | 0.9962 |
|  | 12 | 0.5378 | 0.1171 | 0.3291 | 0.9965 |
|  | 13 | 1.3065 | 0.2827 | 0.2772 | 0.9946 |
|  | 14 | 1.5167 | 0.3166 | 0.2678 | 0.991 |
